# Clinical characteristics of child and adolescent psychiatric outpatients engaging in fireplay or arson: a case–control study

**DOI:** 10.1186/s13034-023-00666-z

**Published:** 2023-10-14

**Authors:** Yoshinori Sasaki, Yuki Hakosima, Kumi Inazaki, Yuki Mizumoto, Takayuki Okada, Katsunaka Mikami, Noa Tsujii, Masahide Usami

**Affiliations:** 1 Department of Psychiatry and Behavioral Science, Tokyo Medical and Dental University Graduate School, Tokyo, Japan; 2 Department of Child and Adolescent Psychiatry, Kohnodai Hospital, National Center for Global Health and Medicine, 1-7-1 Kohnodai, Ichikawa, Chiba 272-8516 Japan; 3 Department of Psychiatry, Tokai University School of Medicine, Kanagawa, Japan; 4 Department of Child Mental Health and Development, Toyama University Hospital, Toyama, Japan

**Keywords:** Fire-related behavior, Child and adolescent psychiatric patients, Attention-deficit hyperactivity disorder (ADHD), Antisocial behavior

## Abstract

**Background:**

Fireplay and arson incidents among children and adolescents have gained attention because of their potentially severe consequences and societal impacts. Understanding the underlying psychiatric characteristics of individuals engaging in fireplay or arson is crucial for early identification and targeted intervention. However, there is a lack of research conducted in clinical psychiatric populations in this context. This study compared the clinical characteristics of child and adolescent psychiatric outpatients who engaged in fireplay or arson with those without such behaviors.

**Methods:**

A retrospective case–control study was conducted using data collected from patients who visited the Department of Child and Adolescent Psychiatry at Kohnodai Hospital, National Center for Global Health and Medicine in Japan, between April 2014 and March 2022. Medical records were checked to see if the patient had practically committed behaviors that corresponded to fireplay or arson. The case group was identified using this process. After identifying the case and control groups, sex, diagnosis, antisocial behavior, abuse history, and children-to-parent violence were assessed and compared by careful review of medical records.

**Results:**

The study identified 64 patients who engaged in fireplay or arson, representing approximately 1.1% of the total 5,587 patients (case group). The median age of the patients’ first fire-related behavior was 13 years (range, 6–18 years). In the case group, 14.1% of the cases involved arson, resulting in substantial damage. Of the remaining 5523 patients, 2268 patients had datasets for the first consultation (control group). The most prevalent diagnosis in the case group was attention-deficit hyperactivity disorder (ADHD), present in 57.8% of the cases. The study revealed a significant association between fire-related behaviors and ADHD as well as antisocial behavior. Gender differences were observed, with boys being more likely to engage in fireplay or arson than girls.

**Conclusions:**

This study suggests that clinicians and mental health professionals should closely consider male sex, ADHD, and antisocial behaviors as potential risk factors for fire-related behaviors. Monitoring the case group for the development of psychiatric disorders, including the use of illegal drugs, is recommended to prevent future arson incidents.

## Background

Fireplay and arson incidents among children and adolescents have gained considerable attention because of their potentially severe consequences and societal impacts. A recent study found that fireplay accounted for 42% of injuries and 62% of deaths in children aged 0–4 years. Furthermore, 84% of fireplay-related injuries were caused by children playing with matches or lighters [1]. Children exhibiting fireplay and fire-setting behaviors often display various behavioral and psychological problems. In samples of inpatient and hospitalized individuals, fire-setters and children engaging in fireplay were characterized by aggression, hostility, delinquency, and cruelty [2, 3]. Numerous studies comparing children based on fire-setting status (non, minor, and severe) have consistently shown higher levels of poor social skills and social judgment among those with fire-setting behavior [3–6]. Additionally, children with fire-setting behaviors tended to demonstrate more internalizing behaviors than their peers. For instance, Martin et al. [7] found that fire-setters reported more suicidal thoughts than their peers who did not show such behaviors. Another study comparing juvenile arsonists and criminals revealed that 74% of arsonists reported suicidal thoughts, with 44% reporting having attempted suicide [8]. Cognitive, academic, and attentional characteristics differentiate children and adolescents who set fires from their peers without such behaviors. Children who engage in fireplay are associated with poor planning abilities and a limited understanding of cause-and-effect relationships. The fire-setters and delinquent controls showed signs of “poor academic performance, a history of grade failure, and truancy” [9].

Age and gender have also consistently emerged as significant predictors of fire-setting behavior, with boys of all ages being more prone to setting fires than girls. Numerous studies have reported higher prevalence rates in boys, ranging from 69 to 91% in certain samples [2, 4, 7, 8, 10–12]. Boys were also more likely to have engaged in repeated fire-setting incidents in another study [9]. Regarding the age of children, it has been linked to the type of fire-setting behavior exhibited, with fireplay observed to correspond to specific developmental age ranges. Typically, interest in fire emerges in children between the ages of three and five years. Fire-setting behavior at this age may not be a cause for immediate concern as it can be part of a child’s natural curiosity [13]. Clinical studies of juvenile fire-setters have confirmed that many children experience their first fire between the ages of six and eight [5, 14]. An older age is associated with a higher likelihood of seeking ignition materials and repeating this behavior [12]. Additionally, a study focusing on 182 fire-setting children and adolescents referred to the New Zealand Fire Awareness and Intervention Program over a 10-year follow-up period showed that these children had a high rate of recidivism [15].

Understanding the underlying psychiatric characteristics of individuals who engage in fireplay or arson is crucial for the early identification and application of targeted interventions to mitigate potential harm. Another characteristic that is shown to have a high prevalence among children who engage in fire-setting behaviors is attention-deficit hyperactivity disorder (ADHD). For example, a fire-setter intervention program found that approximately 20% (115/579) of youth fire-setters were diagnosed with ADHD [16]. Data from 182 fire-setting children and adolescents provided by the New Zealand Fire Service revealed that family stress and a diagnosis of ADHD were associated with previous fire-setting behavior [15]. Maltreated children are also more likely to become involved in setting fires out of anger [17]. Research suggests that the associated impulsivity plays a role in their inability to inhibit their behavior and contributes to their involvement in playing with lighters, matches, and fire-setting. When comparing fire-setters and non-fire-setters, juvenile fire-setters with impulsive behavior exhibited less inhibition compared to non-fire-setters in residential placements [5]. Furthermore, fire-setters and children who play with matches have been rated higher in terms of “emotionality, impulsivity, and lower sociability” compared to non-fire-setters [2]. Impulsivity also differs by fire-setting groups based on severity, with more severe and persistent fire-setters displaying higher levels of impulsivity [8]. Fire setting is a serious form of antisocial behavior that is strongly associated with conduct disorder [18, 19]. Despite these descriptions, there is a lack of research conducted in clinical children and adolescent psychiatric populations involved in fire-related behaviors, and a poor understanding of the specific clinical profiles and developmental trajectories of these children and adolescents.

Therefore, this study aimed to compare the clinical characteristics of children and adolescent psychiatric outpatients who engaged in fireplay or arson with those of child and adolescent patients who did not engage in such behaviors. Previous research suggests that those involved in fire-related behaviors are more likely to be boys, have a history of abuse, and diagnosis of ADHD and antisocial behavior. In this case–control study, we examined the prevalence of psychiatric disorders and related risk factors in the patients. The findings of this study have important implications for early identification, intervention, and prevention strategies aimed at individuals at risk of fireplay and arson. By identifying the psychiatric profiles and risk factors associated with these behaviors, clinicians and mental health professionals can develop customized interventions and support services for the affected individuals and their families.

## Methods

### Study setting

The participants were patients who visited the Department of Child and Adolescent Psychiatry, Kohnodai Hospital, National Center for Global Health and Medicine in Japan between April 2014 and March 2022. The patients were younger than 15 years of age at the initial visit. Psychologists and psychiatrists managed the initial interview forms, which included patients’ demographic and clinical characteristics. Psychiatrists specializing in child and adolescent psychiatry diagnosed and treated all patients according to the Diagnostic and Statistical Manual of Mental Disorders, Fifth Edition (DSM-5) [20].

This study regarded patients who engaged in illegal activities, such as smoking, drinking, or violence and inflicting bodily injury, as having antisocial behavior because smoking and drinking are legal in Japan after the age of 20 years. In the initial consultation, the psychiatrist in charge examined and assessed patients and determined whether the patients had any reports of having experienced abuse (e.g., neglect, psychological, physical, and sexual abuse). The Department works with local child protection services; therefore, information about abused children is often available prior to the initial consultation. In addition, in-charge psychiatrists interviewed parents and patients separately to identify abused children, as needed. Children-to-parent violence was defined as continuous and repetitive physical violence toward parents with whom the children lived or damage to the property. This definition did not include psychological violence such as the use of abusive words, nor sexual abuse [21]. Patients with moderate-to-severe intellectual disability according to the DSM-5, organic brain disease, drug-induced psychiatric disease, traumatic brain injury, or genetic syndromes were usually referred to other medical institutions prior to the initial consultation. Thus, these patients were all excluded from the study sample.

### Study design

A retrospective case–control design was used to evaluate the clinical characteristics of children and adolescent psychiatric patients who played with fire or committed arson.

Kohnodai Hospital had a system to search for words in all medical records to detect words related to fireplay or arson over an 8-year period (i.e., between April 2014 and March 2022). After the Japanese word for “fire” was entered into the search system, the relevant medical records were detected, and thus were checked individually to assess whether the patients had practically committed behaviors that corresponded to fireplay or arson. The case group was identified using this process. The control group was established based on the registered datasets of patients who visited the Department of Child and Adolescent Psychiatry at Kohnodai Hospital for the first time between April 2014 and March 2022. After identifying case and control groups, sex, diagnosis, antisocial behavior, abuse history (i.e., neglect, psychological, physical and sexual abuse), and children-to-parent violence were assessed and compared by carefully reviewing the medical records.

### Statistical analyses

Fisher’s exact test was used to compare the proportions of binary variables between the two groups (primary outcome). All statistical tests were two-tailed, and p < 0.05 were considered statistically significant. Those with a significant difference (p < 0.05) in terms of the primary outcome were included in the multivariate logistic regression analysis (secondary outcome). Analyses were performed using Easy R Package version 1.40 [22].

## Results

### Participants and descriptive data

**Fig. 1:**
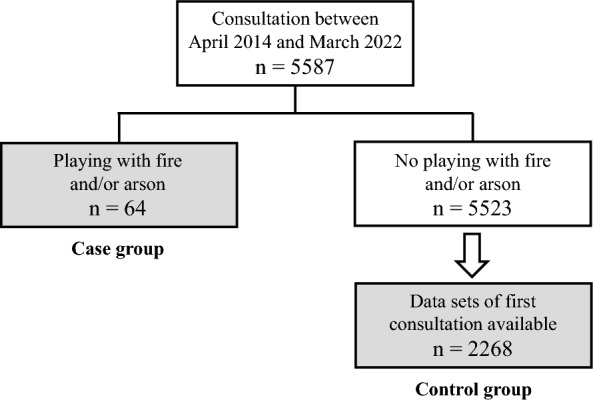
Flowchart for case and control groups

A total of 5587 patients had a consultation in Kohnodai Hospital between April 2014 and March 2022. By investigating their medical records, 64 patients who played with fire or committed arson were identified (case group), representing approximately 1.1% of total patients. The age of patients at the time of the first entry in the medical record regarding fire-related behaviors ranged from 6 to 18 years, with a median age of 13 years and a mean age of 12.2 ± 2.5 years. There were nine arson-level cases (14.1%) in which substantial damage had occurred, specifically in beds and bedding, other people’s clothing and books, and bonfires at home. Of the remaining 5,523 patients, 2,268 patients had complete datasets for the first consultation and made up the control group, allowing for the case–control study to be conducted (Fig. 1).

### Outcome data

Table 1 shows the diagnoses of the case group. Several patients had multiple diagnoses. The most common diagnosis was ADHD, with 37 patients, representing 57.8% of the case group. The second most common diagnosis was autism spectrum disorder (ASD), with 34 patients, representing 53.1% of the case group. The third most common diagnosis was intellectual disability, with 5 patients (only mild intellectual disability) representing 7.8% of the case group. Some examples of antisocial behaviors in the case group were shoplifting, theft, property damage, sexual assault, violence and inflicting bodily injury. No patients were found who might have used illegal drugs (Table 1).

**Table 1:** Diagnoses of the case group

Diagnosis	% (n = 64)
Attention-Deficit Hyperactivity Disorder (ADHD)	57.8 (n = 37)
Autism Spectrum Disorder (ASD)	53.1 (n = 34)
Intellectual disability	7.8 (n = 5)
Oppositional Defiant Disorder (ODD)	3.1 (n = 2)
Reactive attachment disorder	3.1 (n = 2)
Trichotillomania	3.1 (n = 2)
Posttraumatic Stress Disorder (PTSD)	3.1 (n = 2)
Specific learning disorder	3.1 (n = 2)
Other diagnosis^a^	1.6 (n = 1)

^a^ “Other diagnosis” included persistent depressive disorder, brief psychotic disorder, obsessive–compulsive disorder, conduct disorder, major depressive disorder, kleptomania, social anxiety disorder, insomnia disorder, specific phobia, other specified anxiety disorders, and schizophrenia

The clinical characteristics of the participants are presented in Table 2. The proportions of boys with ADHD, ASD, antisocial behavior, abuse history, and child-to-parent violence were significantly higher in the case group than in the control group. No significant differences were observed in the proportions of intellectual disability.

**Table 2:** Clinical characteristics of the participants

	Case % (n = 64)	Control % (n = 2268)	OR	95% CI	P value
Male sex	85.9 (n = 55)	57.2 (n = 1298)	4.56	2.22–10.56	** < 0.01**
ADHD	57.8 (n = 37)	16.9 (n = 383)	6.74	3.94–11.65	** < 0.01**
ASD	53.1 (n = 34)	40.1 (n = 910)	1.69	1.00–2.88	**0.039**
Intellectual Disability	7.8 (n = 5)	10.7 (n = 242)	0.71	0.22–1.78	0.68
Antisocial behavior	46.9 (n = 30)	12.3 (n = 279)	6.28	3.65–10.76	** < 0.01**
Abuse history	21.9 (n = 14)	11.2 (n = 254)	2.22	1.12–4.15	**0.02**
Child-to-parent violence	29.7 (n = 19)	10.6 (n = 240)	3.56	1.94–6.34	** < 0.01**

ADHD, attention-deficit hyperactivity disorder; ASD, autism spectrum disorder

Statistically significant values are presented in bold

Table 3 shows the results of multivariate logistic regression analyses adjusted for sex, ADHD, ASD, antisocial behavior, abuse history, and child-to-parent violence, which were statistically significant (see Table 2). The findings showed that sex, ADHD, and antisocial behavior were independently associated with behaviors related to fireplay or arson after adjusting for six parameters.

**Table 3:** Multivariate logistic regression analyses

	OR	95% CI	P value
Male sex	2.39	1.14–5.02	**0.02**
ADHD	4.92	2.88–8.43	** < 0.01**
ASD	1.61	0.94–2.74	0.08
Antisocial behavior	3.54	1.99–6.29	** < 0.01**
Abuse history	1.10	0.56–2.18	0.78
Child-to-parent violence	1.82	0.99–3.37	0.06

ADHD, attention-deficit hyperactivity disorder; ASD, Autism spectrum disorder

Each parameter was adjusted for sex, ADHD, ASD, antisocial behavior, abuse history and child-to-parent violence

Statistically significant values are presented in bold

## Discussion

We evaluated the clinical characteristics of children and adolescent psychiatric patients who had played with fire or had committed arson. The hypothesis that they are more likely to be boys, have a history of abuse, have a diagnosis of ADHD, and antisocial behavior was partly supported.

### Boys

Among the case groups, the age at the time of hospital consultation following the incident ranged from 6 to 18 years, with a mean of 12.4 years. Kolko investigated 307 children (aged 6–13 years) from nonpatient, outpatient, and inpatient samples who were classified into fire-setters, match-players, and non-fire-setter groups [2]. This cited study showed that children and adolescent psychiatric patients were more likely to be boys, even at an older age. Moreover, in another study, female juvenile arsonists were reported to be more likely to be experienceing family stress, while male juvenile arsonists were more likely to be involved with delinquent groups [23]. These studies showcase that more in-depth findings could have been obtained in the current research had we examined the case group in terms of family stress and involvement with delinquent groups. Future researchers are suggested to conduct such examinations.

### ADHD

Fireplay and arson were strongly associated with ADHD in this study. The association between ADHD and fire-related behavior was shown not only in community populations, as shown in previous studies [15, 16], but also in the clinical population of children and adolescents in a psychiatry department. It is possible that the impulsivity specific to ADHD is more associated with fire-related behaviors than the restricted interests specific to ASD. Previous studies in community populations have shown that family support, such as living with both parents, decreases the probability of engaging in fire-setting behavior [15]. Therefore, examining the environment of patients with ADHD in the clinical population under scrutiny in the current study might lead to important insights as to ways to improve fire-related behaviors. Since the impulsivity and vigilance deficits that characterize ADHD might place these children at higher risk for specific types of burn injuries (vs. children without ADHD) [24], it might be clinically useful to examine children and adolescents with the characteristics similar to those of the case group of this study for burn injuries.

### Abuse

There was no statistically significant difference in the percentage of abuse history between the case and control groups after multivariate logistic regression analysis. Previous studies have shown a relationship between fire-related behaviors and a history of abuse in community populations and healthy youth [14]. These differences in the results of the cited study and the current study may be attributed to the fact that both the case and control groups in this study were children and adolescent psychiatric patients. Stress from parents due to abuse was reported to motivate fire-setting [17], albeit the children and adolescent psychiatric patients in this study were participants who went to consultations in the hospital because of stress at school, at home, or in relationships with parents or friends. Moreover, both the case and control groups had a certain percentage of abuse history, regardless of the presence of fire-related behaviors. Therefore, the proportion of patients with a history of abuse did not differ significantly between the case and control groups.

### Antisocial behavior

The association between antisocial behavior and fire-setting behavior was shown not only in the community populations of previous studies [18, 19], but also in the clinical settings shown in the present study. Previous studies have shown a link between fire-setting behaviors and conduct disorder, underpinning that participants in this study may have a likelihood of developing conduct disorder as they age. Because fire setting and other antisocial behaviors increase the risk of lifetime and current psychiatric disorders, even in the absence of a DSM-IV diagnosis of antisocial personality disorder [25], it might be necessary to follow children with antisocial behaviors to prevent the development of antisocial personality disorder.

### Overall

Several psychiatric studies have been conducted on adults involved in arson. Psychiatric diagnoses commonly associated with arson include schizophrenia, bipolar disorder, substance use disorders, mood disorders, anxiety disorders, intermittent explosive disorder, pervasive developmental disorders, attention deficit hyperactivity disorder, and intellectual disability [26, 27]. However, it is important to note that not all adults with psychiatric disorders commit arson, and other factors such as personality disorders and environmental causes may also play a role [28, 29]. Some studies have suggested that there is a pathway for children with ADHD through oppositional defiant disorder and conduct disorder to antisocial personality disorder [30, 31]. ADHD and antisocial behavior were also identified as significant factors associated with fire-related behaviors for the case group in this study, even after multivariate analysis. Therefore, the case group of this study, and children and adolescents with similar characteristics, needs to be monitored to ensure that these children and adolescents do not develop psychiatric disorders, including the use of illegal drugs that lead to arson in adulthood. There is limited research that verifies the association between arson committed by adult psychiatric patients and childhood psychiatric disorders. However, this study provides some insights regarding the link between patients diagnosed with ADHD and antisocial behaviors in childhood and the likelihood of it leading to their committing arson as adult psychiatric patients later in life.

### Study limitations

This study has several limitations that must be considered. First, a measurement bias may have existed for several reasons. Ascertainment bias affected this study, as it could not completely define fireplay or arson. Moreover, reporting bias was present in this study. Not all psychologists and psychiatrists in charge at the hospital actively asked all patients about engagement in fire-related behaviors, and since most data on fireplay or arson were based on the system used to search for words in all medical records, this might have led to missing information or patients who were involved in fireplay or arson. Furthermore, most data of the control group were registered after the initial consultation. Thus, it is possible that additional information regarding substance and child abuse could have been obtained in case further examinations were possible. Second, this study cannot be considered representative of children and adolescent psychiatric patients involved in fireplay or arson because it was conducted in a single hospital. Moreover, and as mentioned in the Methods section, some psychiatric disorders, such as drug-induced psychiatric disease, were excluded from the study prior to the initial consultation; this introduced selection bias to the sample and hindered generalizability. Finally, the results of this study confirmed the associations between fire-related behaviors and the clinical characteristics of children and adolescent psychiatric patients, but could not determine causal relationships.

## Conclusions

This study compared the clinical characteristics of children and adolescent psychiatric outpatients who engaged in fireplay or arson with those of children and adolescent who did not engage in such behaviors. The study found a significant association between fire-related behaviors and ADHD as well as antisocial behavior. Boys were more likely to engage in fireplay or arson than girls. These findings highlight the importance of early identification and intervention in child and adolescent psychiatric outpatients at risk of fireplay and arson. They also suggest that clinicians and mental health professionals consider the male sex, ADHD, and antisocial behavior as potential risk factors. Monitoring children and adolescents with characteristics that resemble those of the case group of this study for the development of future psychiatric disorders, including the use of illegal drugs, is recommended to prevent future arson incidents.

## Data Availability

The data that support the findings of this study are available from the Ethical Committee of the National Center for Global Health and Medicine in Japan through the email address rinrijm@hosp.ncgm.go.jp upon reasonable request and with permission. Nonetheless, restrictions apply to the availability of these data, which were used under a license for the current study and are not publicly available.

## Abbreviations

ADHD: Attention-deficit/hyperactivity disorder
ASD: Autism spectrum disorder
DSM-5: Diagnostic and Statistical Manual of Mental Disorders, Fifth Edition
